# Determination of Sulfonamide Residues in Food by Capillary Zone Electrophoresis with On-Line Chemiluminescence Detection Based on an Ag(III) Complex

**DOI:** 10.3390/ijms18061286

**Published:** 2017-06-16

**Authors:** Tingting Dai, Jie Duan, Xinghua Li, Xiangdong Xu, Hongmei Shi, Weijun Kang

**Affiliations:** School of public Health, Hebei Medical University, Shijiazhuang 050017, China; daitingting2320@163.com (T.D.); duan224462@163.com (J.D.); LIXH026@163.com (X.L.); Xuxd@hebmu.edu.cn (X.X.)

**Keywords:** capillary electrophoresis, chemiluminescence, Ag(III) complex, sulfonamides, determination

## Abstract

The presence of sulfonamide (SA) residues in foods is largely due to the raising of animals with sulfonamide antibiotics added or polluted feedstuff. In this paper, a sensitive method was developed for the determination of the residues of three sulfonamides in animal-derived food; the SAs include sulfadimidine (SDD), sulfadiazine (SDZ), and sulfathiazole (STZ). The method is based on capillary zone electrophoresis (CE) with online chemiluminescence (CL) detection, using an Ag(III) complex as an oxidant. These SAs have an inhibiting effect on the Ag(III)–luminol CL reaction. The electrophoretic buffer is 12.0 mM sodium borate. Under a set of optimized conditions, the linear ranges for the detections were found to be 10.0–200 µg·mL^−1^ for SDD and SDZ, and 2.0–50.0 µg·mL^−1^ for STZ. The detection limits were 2.75, 3.14, and 0.65 µg·mL^−1^ for SDD, SDZ, and STZ, respectively. Relative standard deviations (RSD) for the peak heights were between 2.1% and 2.8% (*n* = 7). The proposed method was used in the analysis of the SAs in samples from pork meat, chicken meat, and milk, showing satisfactory detection results. A reaction mechanism was also proposed for the Ag(III)–luminol–SA CL reactions. The method has potential applications for the monitoring of residue levels of the three SAs in food, providing food safety data.

## 1. Introduction

As a large group of synthetic antibiotics, sulfonamides (SAs), are commonly used in animal feedstuff and fish cultures. The high efficiency and relatively low cost of SAs have stimulated their ubiquitous utilization in veterinary practices for prophylactic and therapeutic purposes. The presence of SA residues in food intended for human consumption is a great concern since SAs are potentially carcinogenic, possibly producing antibiotic resistance and allergic reactions for humans [1,2]. Sulfadimidine (SDD), sulfadiazine (SDZ), and sulfathiazole (STZ) are the most common of SA drugs (structures are given in Figure S1), and have a broad spectrum of antibacterial activity. SDZ is used in a variety of animal-sensitive bacterial infections; it is absorbed after oral administration and undergoes a slow excretion [3]. SDD is absorbed rapidly and completely after oral administration and an effective concentration remains in the blood for a long time [4]. STZ has a high tendency to bind with plasma proteins and a high degree of acetylation in vivo. On other hand, STZ has a low solubility and is prone to crystallization, causing damages in the urine system and kidneys [5]. Animal-derived food with these SA drug residues may cause some diseases in humans including aplastic anemia and agranulocytosis. In the last decade, increasing attention has been paid to food safety, and food containing SAs are certainly and closely related to food safety concerns. However, the quantification of veterinary drug residues in food encounters some difficulties due to their low content. Thus, to establish a reliable and a low-cost detection method that is also sensitive and has a fast response for the determination of SA drug residues is of critical importance.

For detecting SAs in food, the analytical technique most widely applied is high-performance liquid chromatography (HPLC), which is combined with different detectors [6,7] including mass spectrometry (MS) [8,9], ultra-violet (UV) [10,11], and fluorescent [12,13] and diode array spectrometers [14].

In recent years, capillary electrophoresis (CE) has proven to be a powerful separation technique and has achieved similar sensitivity, selectivity, and specificity as HPLC. The relatively high efficiency, rapid analysis time, and small consumption of both solvent and sample make CE an environmentally friendly detection technique. Consequently, it is widely applied to the analysis of diverse samples [15,16]. Several kinds of detectors for CE, including UV [17], laser-induced fluorescence (LIF) [18], and MS [19], have been used for the analysis of SA-containing samples. UV detection is the most common approach adopted by commercial CE systems because of its general applicability; in these systems, the optical path length is however very limited due to the very small inner diameter (i.d.) of the capillary, resulting in a sensitivity limit. LIF detectors for CE certainly require the derivatization of analytes to improve their fluorescent properties. Although the MS detection technique has a great advantage for the analysis of complex samples, a much higher cost of running the MS instrument limits its high throughput. Chemiluminescence (CL) detection systems, known to have a very sensitive detection nature, have been developed for CE, and have been applied to the analysis of complex matrices such as clinical medicine and food [20,21]. Luminol-oxidant systems are the most frequently used CL reactions, for instance, luminol-H_2_O_2_ [22], luminol-H_2_O_2_-horseradish peroxidase [23], luminol-Fe(III) [24], and luminol-H_2_O_2_-Cu(II) [25] have been reported. So far, the determination of SAs by the CE coupled with CL detector technique (CE–CL) has not been found.

Over the last few years, part of our research effort has been devoted to the study of oxidation reactions by an Ag(III) complex anion ([Ag(HIO_6_)_2_]^5−^) [26,27,28]. [Ag(HIO_6_)_2_]^5−^, possessing a square-planner geometry around the metal center (the structure is illustrated in Figure S2), is fairly stable in alkaline media, and has a strong oxidation capability [29,30,31]. Oxidation of luminol by the Ag(III) complex can generate CL signals; based on the enhancement or inhibition of the signals by some analytes, the Ag(III)-luminol CL detection system coupled with a flow-injection has been used successfully in the determination of biological samples including cortisol [32], dopamine [33], and moreover high sensitivities have been obtained in these determinations. The Ag(III)-luminol system was also utilized as a detector in CE and HPLC, conferring a new methodology for the determination of some antioxidants and catecholamines [34,35]. In this work, we report the utilization of the luminol–Ag(III) system as a detector for CE and the employment of the CE–CL setup for the determination of SDD, SDZ, and STZ in animal-derived food. Moreover, a reasonable CL reaction mechanism is proposed.

## 2. Results

### 2.1. Optimization of the CL Detection System

**Figure 1:**
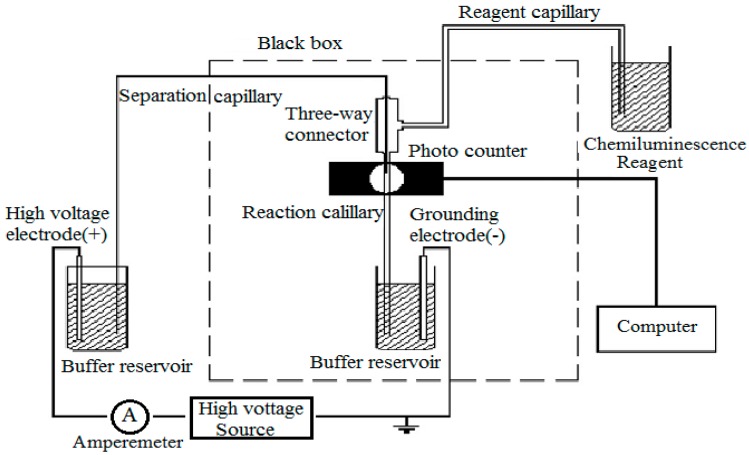
Diagram of the laboratory-built CE–CL setup.

In the Ag(III)–luminol detection system, the CL emission is produced by the oxidation of luminol with the Ag(III) complex in alkaline media while SAs in samples can inhibit the CL intensity. Figure 1 illustrates the laboratory-built CE–CL system, in which luminol migrated from the separation capillary (i.d. = 50 μm) and the Ag(III) solution was delivered from the reagent capillary (i.d. = 200 μm) at the detection window. The pH value of the CL reaction mixture in the reaction capillary (i.d. = 530 μm) is dependent primarily on the content of NaOH in the Ag(III) solution.

Major parameters including the concentrations of luminol, Ag(III) complex, and hydroxide which could influence the sensitivity of the detection system were optimized in order to maximize the sensitivity. The influence of the luminol concentration on the CL intensity was investigated between 1.0 and 4.0 mM, keeping [Ag(III)] = 0.05 mM, [NaOH] = 10 mM, and sodium borate at a concentration of 12.0 mM (pH 9.0). The results showed that the maximum emission could reach at [luminol] = 1.50 mM (shown in Figure S3). Subsequently, the variation of [Ag(III)] was studied in the range of 0.010–0.10 mM while [luminol] = 1.50 mM remained constant. Clearly, [Ag(III)] = 0.06 mM was the optimized concentration (shown in Figure S4).

The change of [NaOH] was found to have a large influence on the intensity of the CL emissions. The [NaOH] in the Ag(III) solution was investigated between 3.0 and 30.0 mM. The CL emission versus [NaOH] profile shown in Figure S5 demonstrates that the best inhibited CL signal was obtained when [NaOH] = 15.0 mM, corresponding to a pH of about 12.3.

### 2.2. Optimization of Capillary Electrophoresis (CE) Separation Conditions in the Presence of SAs

#### 2.2.1. Selection of CE Buffer Solution and Its pH Value

In the CE–CL detection system, the sample solutions containing SDD, SDZ, and STZ were delivered by gravity through the separation capillary. Sodium borate buffer concentrations and pH values (adjusted by NaOH solution) had an influence on the CE separation efficiency and the intensity of CL signals. To examine these effects, the concentration of sodium borate buffer was varied between 2.0 and 18.0 mM. Results showed that both the migratory rate and the CL signals of the three SAs were clearly affected by the change of buffer concentrations; moreover, the signal intensity was inversely proportional to the buffer concentration. In consideration of the separation efficiencies and the CL signal intensities of the three SAs, the optimized buffer concentration was settled at 12.0 mM. The pH of the running buffer at about 9.5 gave a good separation of SDD, SDZ, and STZ and the total separation was completed in less than 12 min.

#### 2.2.2. Selection of Applied Voltage

Theoretically, the length of the separation capillary and the applied voltage have a strong impact on the migration rate and the CL intensities. When a voltage range of 8 to 20 kV was examined, the CE separation efficiency was proportional to the applied voltage, provided the separation capillary length was fixed. However, the applied voltage could only be changed in a range, because the Joule heating in the capillary was increased when the voltage was increased, while the heating effect could repress the capillary efficiency and viscosity of the running buffer. When the best signal-to-noise ratio was considered, a voltage of 18 kV was the best parameter for acquiring the maximum CL intensity, cf. Figure S6.

#### 2.2.3. Selection of Injection Mode and Injection Time

Gravity injection is a common mode of injection for capillary electrophoresis. The injection time is essentially determined by the volume of samples to be injected. Thus, gravity injection and an injection time of 18 s were chosen for the experiments performed in this work. The optimized conditions for the determination of the three SAs are summarized as: 12.0 mM sodium borate buffer containing 1.5 mM luminol as the running buffer at pH 9.5, 0.06 mM Ag(III) solution containing 15.0 mM NaOH, an applied voltage of 18 kV, and an injection time of 18 s.

### 2.3. Linearity, Limit of Detection, and Reproducibility

Under the above optimized conditions, a series of standard solutions containing the three SAs were detected. The linear ranges, limits of detection (LODs), and relative standard deviations (RSD) were obtained. LOD was calculated based on the relative CL intensities which were three times above the baseline noise. The reproducibility represented by RSD was investigated by injections of the standard solutions of the SAs at 20.0 µg·mL^−1^, each for seven times, and the CL peak heights were recorded conferring the RSD values. Under the optimized detection conditions, the linear ranges were 10.0–200 µg·mL^−1^ for SDD and SDZ, and 2.0–50.0 µg·mL^−1^ for STZ (Standard curves are shown in Figure S7. Figures S8–S10 are electropherograms obtained for the standard solutions of SDD, SDZ and STZ, respectively). The LODs were 2.75, 3.14, and 0.65 µg·mL^−1^ for SDD, SDZ, and STZ, respectively. Relative standard deviations (RSD) for the peak heights were between 2.1% and 2.8% (*n* = 7).

Compared to other analytical techniques, the LOD of the present detection system is lower than or equivalent to those for HPLC-UV (46.9–150.0 µg kg^−1^) [10], HPLC-fluorescence (14.0–85.0 µg kg^−1^) [36], and CE–UV (5–10 µg kg^−1^) [37] systems which have been used for the determination of sulfonamides in food.

### 2.4. Sample Analysis

**Figure 2:**
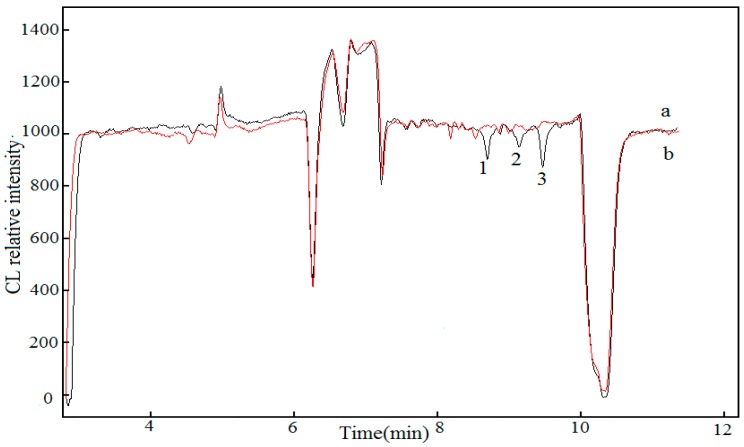
Electropherograms obtained under the optimized conditions for the milk sample (a, red line) and the same sample spiked with standard sulfonamides(SAs) (b, black line). Assignment of peaks: Peak 1, sulfadimidine (SDD); Peak 2, sulfadiazine (SDZ); Peak 3, sulfathiazole (STZ).

**Figure 3:**
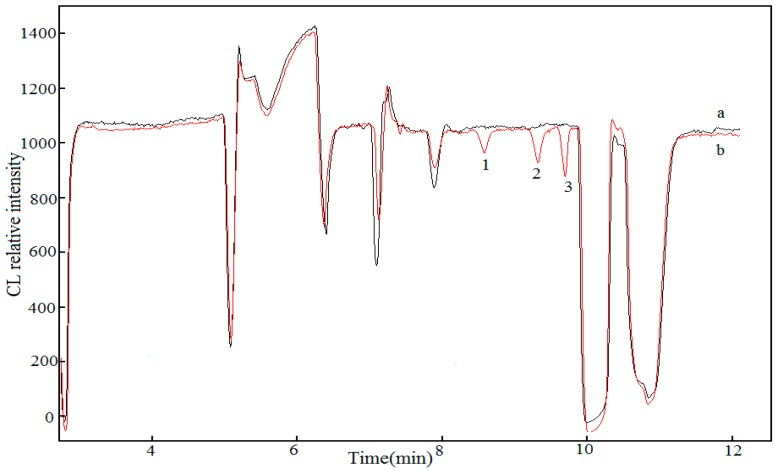
Electropherograms obtained under the optimized conditions for the pork sample (a, black line) and the same sample spiked with the standards of SAs (b, red line). Assignment of peaks: Peak 1, SDD; Peak 2, SDZ; Peak 3, STZ.

**Figure 4:**
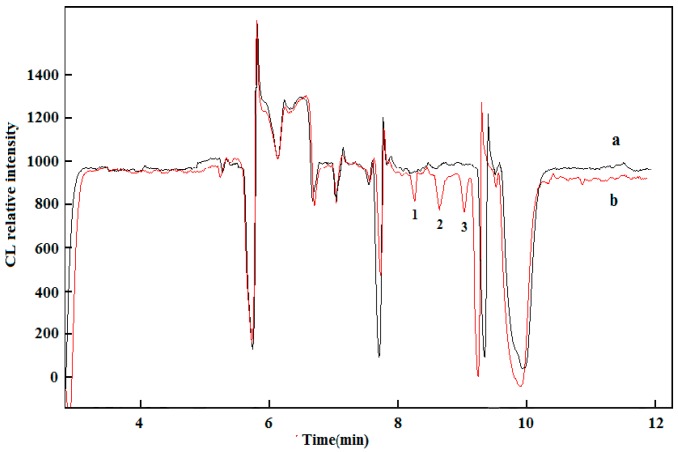
Electropherograms obtained under the optimized conditions for the chicken meat sample (a, black line) and the same sample spiked with the standards of SAs (b, red line). Assignment of peaks: Peak 1, SDD; Peak 2, SDZ; Peak 3, STZ.

The proposed CE–CL method was applied to the determination of SDD, SDZ, and STZ in milk, pork, and chicken. The preparation of the samples is described in the experimental section below. Figure 2, Figure 3 and Figure 4 show electropherograms obtained for the samples (a) and the samples spiked with the three SAs, respectively. Peaks 1–3 were identified as SDD, SDZ, and STZ, respectively, based on the migratory times from their standards. Other peaks observed on the electropherograms may stem from the complex food matrices. The results shown in Figure 2, Figure 3 and Figure 4 indicate that the residues of the three SAs in milk, pork, and chicken were not detectable within the detection limits of the present instrumental setup. This conclusion is consistent with those obtained by HPLC (GB/T 29694-2013, LOD, 5.0 µg·kg^−1^) or HPLC-MS techniques (GB/T 22966-2008, LOD, 1.0 µg·kg^−1^), demonstrating that the two methods for the determination of the three drug residues were not significantly different.

### 2.5. Recovery Experiments

The recoveries of the three SAs were investigated by the addition of standard SAs to the samples in order to evaluate the validity of the proposed method. The recoveries were found to be in a range of 79.5 to 112.4% (Table 1). In the calculation of the recoveries, the residual content of the three SAs was assumed as 0 since they are not detectable in the real samples.

## 3. Discussion

The good solubility and strong oxidation capability of [Ag(HIO_6_)_2_]^5−^ in alkaline media make it a very convenient reagent to be incorporated in luminol CL reactions. Compared with other oxidants which have been used in luminol CL reactions, the Ag(III) complex has some advantages for its utilization in CE: (1) the luminol was in NaOH solution which was directly incorporated in the running buffer; (2) no problems with disruptions of the current were noticed, in contrast to the bubble formation when H_2_O_2_ was used as an oxidant. These characteristics can decrease the detrimental turbulence, giving rise to improved reaction efficiency and sensitivity. Many analytes have been found to have a sensitizing effect on the Ag(III)–luminol CL system, and a new CE–CL analytical method was developed for the determination of catecholamines in urine samples [34]. SAs also have an inhibition ability on the luminol–Ag(III) CL system, making luminol–Ag(III) CL possible as a detector in CE for detecting SAs.

**Figure 5:**
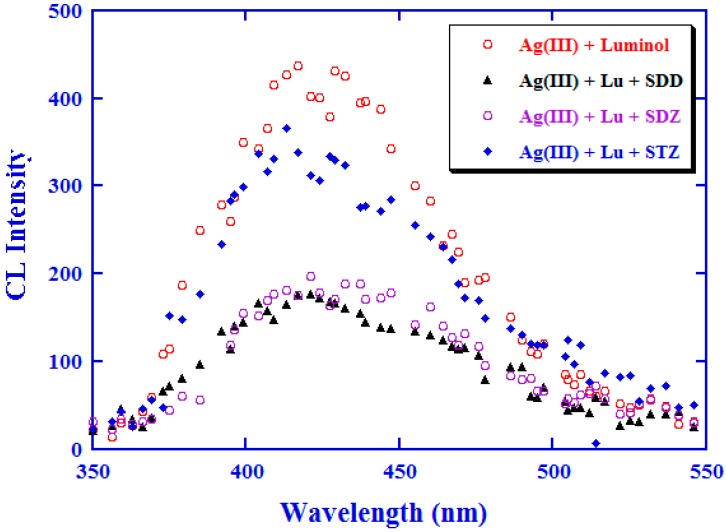
CL spectra of Ag(III)–luminol and Ag(III)–luminol–SA detection systems. Conditions: [Luminol] = 0.10 mM, [SDD] = [SDZ] = 0.2 mM; [STZ] = 0.1 mM; [Ag(III)] = 0.05 mM in 5.0 mM NaOH solution.

The CL reactions of the luminol–Ag(III)–SA systems were also explored by the CL spectra, which were obtained from an F-7000 fluorescence spectrophotometer (Figure 5). The CL spectra obtained for reaction systems of luminol–Ag(III), luminol–Ag(IIII)–SDD, luminol–Ag(IIII)–SDZ, and luminol-Ag(III)–STZ display a common absorption peak at around 425 nm, suggesting that all these CL reactions shared a common emitting species. This emitting species is assumed to be aminophthalate, which is generated from luminol. No doubt the CL intensity of luminol–Ag(III) system is significantly inhibited by the three SAs. On the other hand, STZ is more sensitive than SDD and SD. These spectral results are consistent with the detection limits shown in Table 1.

**Scheme 1:**
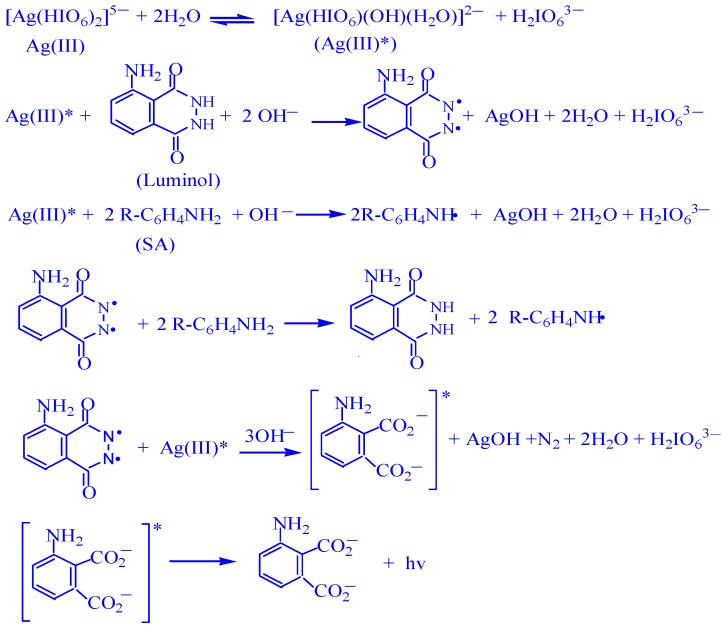
A proposed reaction mechanism for the luminol–Ag(III)–SA CL systems.Symbol “*” denotes active intermediates.

A free radical trapping experiment was also carried. Solutions containing luminol/SDD/SDZ/STZ (2.0 mM and 50 mL) and 8% acrylonitrile were mixed with a 50 mL Ag(III) solution (0.5 mM) in a three-neck flask. The solution mixtures were then flushed with nitrogen gas for 30 min. By stirring the reaction mixture at 40 °C for about 3 h under the protection of nitrogen gas, precipitates appeared. The induction of polymerization of acrylonitrile implies the intervention of free radicals in the reaction course. A reasonable reaction mechanism is proposed and illustrated in Scheme 1 based on the CL spectra and free radical intervention.

The use of SAs in animal feeding is not prohibited in many countries. Animal-derived food samples, such as chicken, pork, and milk, are generally very difficult to be pretreated before analysis. In this work, the Ag(III)–luminol CL reaction system was used as a CE detector and was applied, for the first time, for the determination of three SA residues in milk, pork, and chicken. The above described method might have potential in monitoring the levels of veterinary drug residues for food safety. Furthermore, the proposed reaction mechanism provides a clear base for this methodology. We are currently using the Ag(III)–luminol CL system as a detector for HPLC and CE to analyze samples from different sources [34,35].

## 4. Experiment

### 4.1. Reagents and Solutions

SDD, SDZ, and STZ were purchased from Ehrenstorfer GmbH (Augsburg, Germany). Luminol was obtained from TCI (Tokyo, Japan). AgNO_3_, KIO_4_, K_2_S_2_O_8_, KOH, NaOH, sodium borate, ammonia, hydrochloric acid, perchloric acid, and formic acid were obtained from Sinopharm Chemical Reagent (Shanghai, China) or Tianjin Chemical Reagent Company (Tianjin, China). Methanol and acetonitrile were obtained from Honeywell (Muskegon, MI, USA). All chemicals used in this work were of either analytical grade or chromatographic pure.

Stock solutions of SDD, SDZ, and STZ (2.0 mg·mL^−1^) were prepared by dissolving 50.0 mg of each standard substance in 3.0 mL of 0. 10 M NaOH, which was then diluted to 25 mL with water. Luminol stock solution (20.0 mM) was prepared by dissolving 0.3544 g luminol in 2.8 mL of 1.0 M NaOH, which was diluted to 250 mL with water. [Ag(HIO_6_)_2_]^5−^ was synthesized according to the procedure described in the literature [29]. The Ag(III) concentrations were determined spectrophotometrically at 362 nm using molar absorptivity (*ε* = 1.26 × 10^4^ M^−1^ cm^−1^) [30]. All the above solutions were prepared with ultrapure water, which was made by a Heal Force ultrapure water system (Shanghai Jinghui Water Technologies Corporation, Shanghai, China), and stored in a refrigerator at 4 °C. Working solutions were prepared daily by diluting the stock solutions with water.

### 4.2. CE–CL Detection System and Electrophoresis Procedure

The laboratory-built CE–CL system [34] consisted of: (a) a high voltage power supply (Tianhui Institute of Separation Science, Baoding, China); (b) a photomultiplier tube (PMT, Binsong, Beijing, China); (c) two platinum electrodes; (d) a PEEK (polyetheretherketone) three-way connector that connected the separation capillary, reagent capillary, and reaction capillary (Yongnian Optical Fiber Factory, Handan, China).

The end section of the separation capillary (about 7 cm) was burned to remove the polyimide layer. After etched with hydrofluoric acid for about 50 min, the section was inserted into the reaction capillary which had been scraped to a 1-cm polyimide layer, and used as a detection window. The Ag(III) complex solution was delivered by gravity to the reagent capillary. Luminol migrated from the separation capillary. At the detection window, two solutions were mixed to generate reaction, producing CL signals, which were then amplified by the PMT. The photocurrent was recorded and processed by data acquisition software (Qianpu Software, Shanghai, China).

Before the experiment started, the capillary was firstly activated by flushing with 0.1 M NaOH, followed by pure water for 10 min and then by running buffer (12.0 mM sodium borate containing 2.0 mM luminol, pH 9.5) for about 20 min. Before use, the new capillary was rinsed with 0.1 M NaOH, 0.1 M HCl and water, respectively; each rinse lasted 10 min.

Sample solutions of SAs were introduced into the cathode end of the separation capillary by gravity injection for 18 s at 20 cm height, and was quantified by measuring the relative CL intensity △*I* = *I*_0_ − *I*_t_, where *I*_0_ was the CL background intensity from the luminol-Ag(III) reaction, and *I*_t_ was the CL intensity from the luminol–Ag(III)–SA systems.

### 4.3. Samples Treatment and Extraction Procedure

Pork and chicken meat and milk was bought from the local supermarket. The meat samples were treated according to the Chinese National Standard (GB/T, 29694-2013). A homogenized tissue sample (20 ± 0.05 g) was extracted with 20.0 mL ethyl acetate. The extraction was repeated several times, and extracts were combined into a heart-shaped bottle. After the addition of 4 mL of 0.1 M HCl to the bottle, the liquid in the bottle was evaporated to about 3 mL by rotary evaporation. The heart-shaped bottle was washed with 2 mL of 0.1 M HCl solution and then with 3 mL hexane. The merged solution was shaken by vortex for 30 s, and was then centrifuged at 3000 rpm for 5 min. The hexane layer was discarded, and the lower layer was preserved. A Welchrom P-SCX (3 mL, 500 mg, Shanghai, China) solid phase extraction cartridges (SPE) with a Waters’ extraction vacuum manifold was used as a clean-up and enrichment device for the sample solutions.

The milk sample was treated according to the Chinese National Standard (GB/T 22966-2008). Perchloric acid solution (pH 2.0, 25.0 mL) was added to a milk sample of 20 ± 0.05 mL for extraction. The mixture was shaken by vortex for 1 min, and then sonicated for 10 min. The upper clear layer was purified by SPE Oasis HLB column (hydrophilic-lipophilic-balanced, 6 mL, 500 mg).

### 4.4. Treatment and Application of SPE Column

SCX SPE columns were used as clean-up and enrichment devices for the extracted solutions from pork and chicken meat according to the following steps:

1. Condition: 2 mL methanol followed by 2 mL of 0.10 M HCl.
2. Load: chicken/pork extraction solutions were applied to the cartridges.
3. Wash: 1 mL of 0.10 M HCl, followed by 2 mL of a solvent mixture (50% methanol–50% acetonitrile).
4. Elute: 4 mL of a solution mixture of 5% ammonia–95% methanol (*V*/*V*).

Oasis HLB SPE column was treated and used according to the following steps:

1. Condition: 3 mL of methanol followed by 5 mL of perchloric acid solution (pH, 2.0).
2. Load: the extracted solution from milk was applied to the cartridges.
3. Wash: 5 mL water.
4. Elute: 3 mL methanol.

The elute solution was evaporated by a nitrogen stream at 40 °C, and was reconstituted in 0.5 mL of 0.05 M NaOH solution. All the solutions were filtered through a 0.45-μm syringe filter before CE separation.

## 5. Conclusions

In this paper, we established an analytical system which combined capillary zone electrophoresis with the luminol-[Ag(HIO_6_)_2_]^5−^ chemiluminescence detection; the system was applied successfully to the simultaneous analysis of three sulfonamide residues in pork meat, chicken meat, and milk. The obtained results indicate that the proposed system is a reliable, selective, and sensitive technique. Moreover, the system can deal with the extensive analytical work of the determination of veterinary drug residuals in animal-derived food.

## Figures and Tables

**Table 1:** Recoveries obtained for the three SAs in milk and pork samples.

Sample	Spiked (µg·mL^−1^)			Found (µg·mL^−1^)			Recovery (%, *n* = 3)		
	SDD	SDZ	STZ	SDD	SDZ	STZ	SDD	SDZ	STZ
**Milk**	40.0	40.0	10.0	37.7	40.7	10.6	94.2	101.7	106.0
	60.0	60.0	20.0	51.5	67.4	18.0	85.8	112.4	89.8
	100.0	100.0	40.0	107.2	112.4	35.5	107.2	112.4	88.8
**Pork**	40.0	40.0	10.0	35.2	38.8	8.1	88.1	96.9	80.9
	60.0	60.0	20.0	65.3	65.5	18.6	108.7	109.2	93.2
	100.0	100.0	40.0	112.4	112.4	33.3	112.4	112.4	83.3
**Chicken**	40.0	40.0	10.0	36.8	37.2	7.9	92.3	93.1	79.5
	60.0	60.0	20.0	62.3	64.6	18.2	103.8	107.7	91.3
	100.0	100.0	40.0	108.5	110.5	32.8	108.5	110.5	82.4

## Supplementary Materials

Supplementary materials can be found at www.mdpi.com/1422-0067/18/6/1286/s1.
