# Supplementary material for: Determination of Sulfonamide Residues in Food by Capillary Zone Electrophoresis with On-Line Chemiluminescence Detection Based on an Ag(III) Complex
Source: Int J Mol Sci. 2017 Jun 16;18(6):1286. doi: 10.3390/ijms18061286 (PMC5486108; doi:10.3390/ijms18061286)
Supplement: Supplementary file 1 [file ijms-18-01286-s001.pdf]

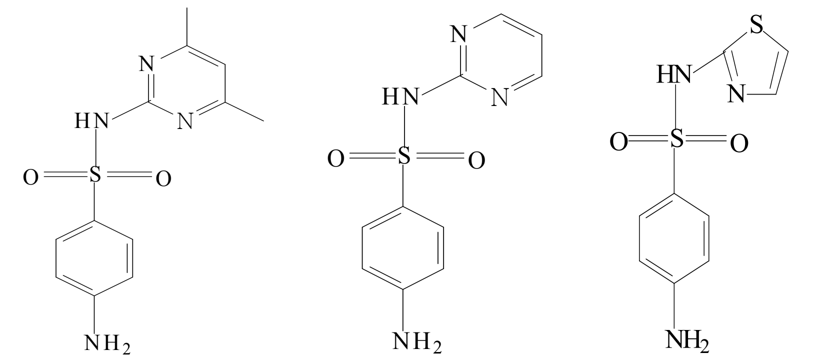

sulfadimidine(SDD) sulfadiazine(SDZ) sulfathiazole(STZ)

Figure S1. Chemical structures of SDD, SDZ and STZ

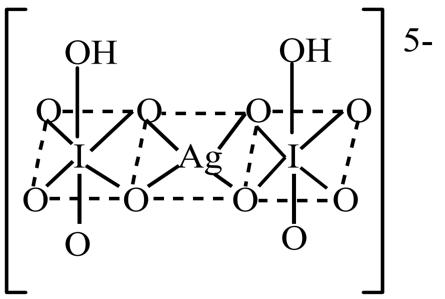

Figure S2. Structure of Ag(III) complex anion $[Ag(HIO_6)_2]^{5-}$

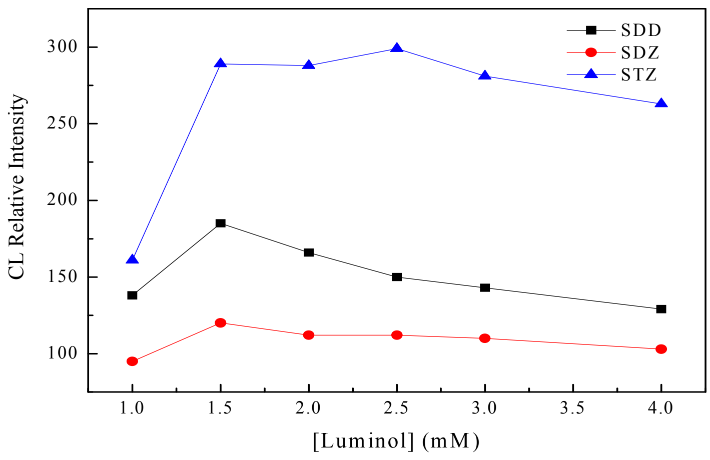

Figure S3. Effect of changing [Luminol] on the CL intensity. Conditions: applied voltage, 18 kV; injection time, 15 s; separation capillary, 60 cm × 50 μm i.d.; running buffer, 12.0 mM sodium borate (pH 9.0); oxidant, 0.05 mM Ag(III) in 10.0 mM NaOH solution; [SDD] = [SDZ] = 50.0 μg mL^{-1}; [STZ] = 20.0 μg mL^{-1}.

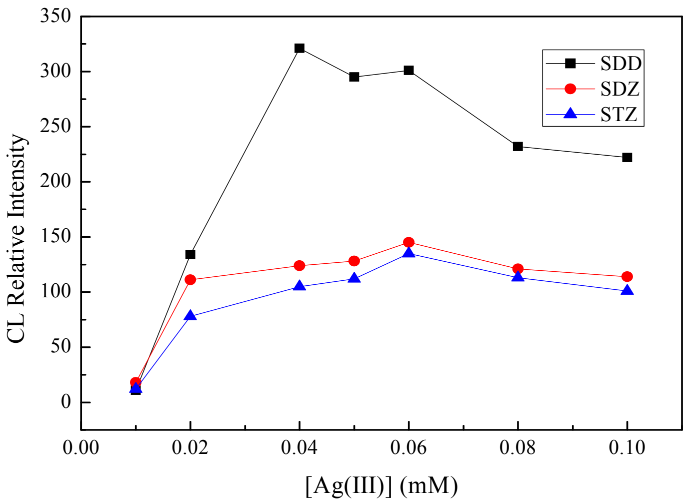

Figure S4. Effect of changing [Ag(III)] on the CL intensity. Conditions: applied voltage, 18 kV; injection time,15s; separation capillary, 60 cm × 50 μm i.d.; running buffer, 2.5 mM luminol in 12.0 mM sodium borate (pH 9.0); oxidant, Ag(III) in 10.0 mM NaOH solution; [SDD] = [SDZ] = 50.0 μg mL^{-1}; [STZ] = 20.0 μg mL^{-1}.

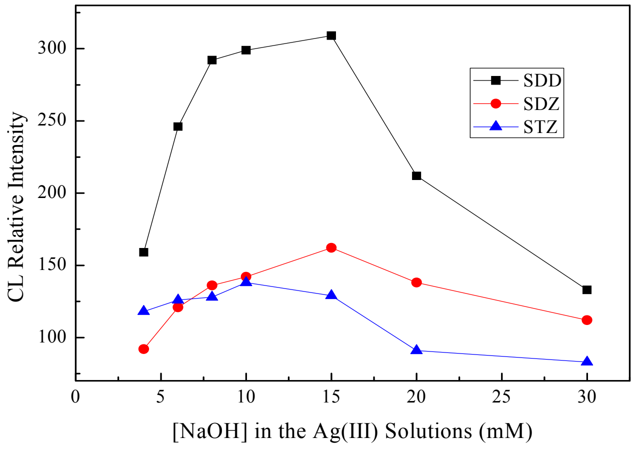

Figure S5. Effect of changing [NaOH] in Ag(III) solution on the CL intensity. Conditions: applied voltage, 18 kV; injection time, 15 s; separation capillary, 60 cm × 50 μm i.d.; running buffer, 1.5 mM luminol in 12.0 mM sodium borate (pH 9.0); oxidant, 0.06 mM Ag(III); [SDD] = [SDZ] = 50.0 μg mL^{-1}; [STZ] = 20.0 μg mL^{-1}.

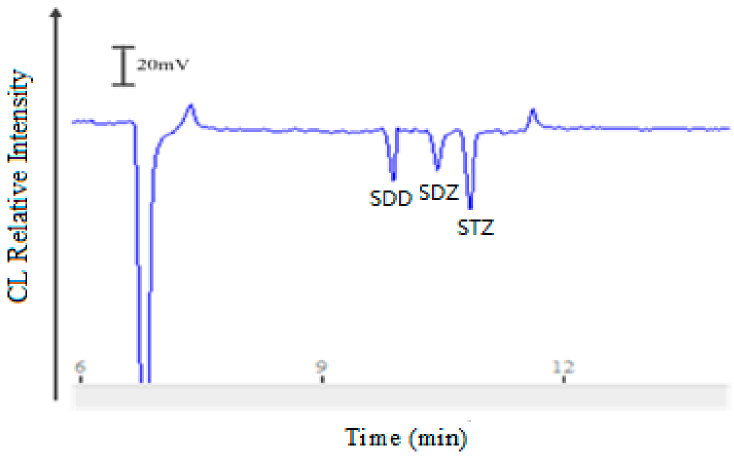

Figure S6. Electropherograms obtained for the standard solutions of sulfonamide(SAs). Conditions: [SDD] = [SDZ] = 50.0 μg mL^{-1}; [STZ] = 20.0 μg mL^{-1}; separation capillary, 60 cm × 50 μm i.d; applied voltage, 18 kV; injection time, 18 s; running buffer, 1.5 mM luminol in 12.0 mM sodium borate (pH 9.5); oxidant, 0.06 mM Ag(III) in 15.0 mM NaOH solution.

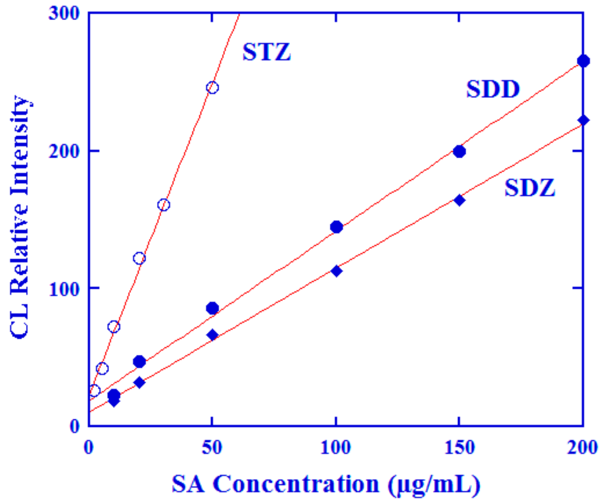

Figure S7. Plot of CL relative intensity versus SA concentration.

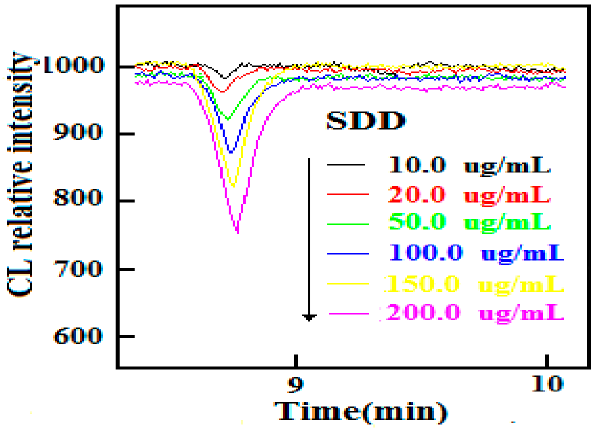

Figure S8. Electropherograms obtained for the standard solutions of SDD. Conditions: separation capillary, 60 cm × 50 μm i.d; applied voltage, 18 kV; injection time, 18 s; running buffer, 1.5 mM luminol in 12.0 mM sodium borate (pH 9.5); oxidant, 0.06 mM Ag(III) in 15.0 mM NaOH solution. Electropherograms of a, b, c, d, e and f, correspond to [SDD] = 10.0, 20.0, 50.0, 100.0, 150.0, 200.0 μg mL^{-1}, respectively.

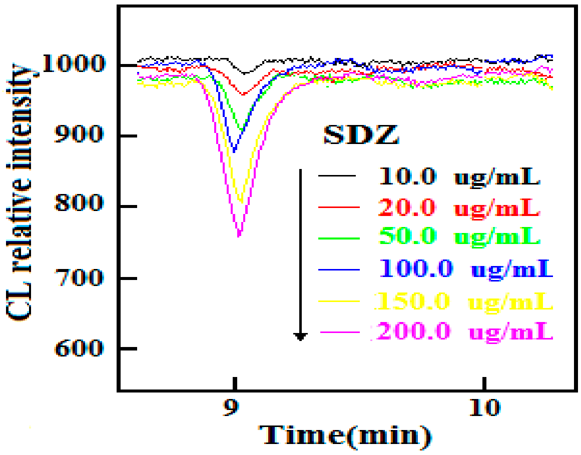

Figure S9. Electropherograms obtained for the standard solutions of SDZ. Conditions: separation capillary, 60 cm × 50 μm i.d; applied voltage, 18 kV; injection time, 18 s; running buffer, 1.5 mM luminol in 12.0 mM sodium borate (pH 9.5); oxidant, 0.06 mM Ag(III) in 15.0 mM NaOH solution. Electropherograms of a, b, c, d, e and f, correspond to [SDZ] = 10.0, 20.0, 50.0, 100.0, 150.0, 200.0 μg mL^{-1}, respectively.

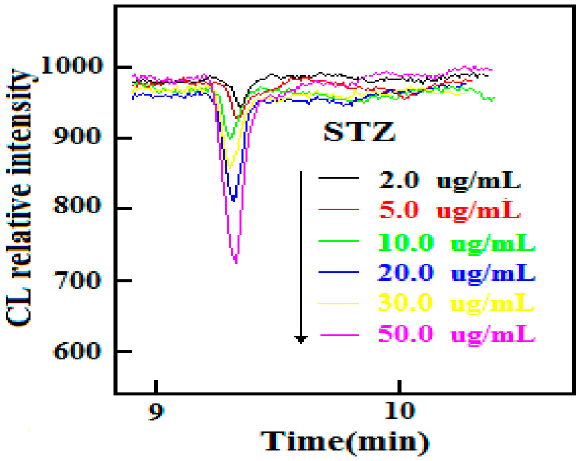

Figure S10. Electropherograms obtained for the standard solutions of STZ. Conditions: separation capillary, 60 cm × 50 µm i.d; applied voltage, 18 kV; injection time, 18 s; running buffer, 1.5 mM luminol in 12.0 mM sodium borate (pH 9.5); oxidant, 0.06 mM Ag(III) in 15.0 mM NaOH solution. Electropherograms of a, b, c, d, e and f, correspond to [STZ] = 2.0, 5.0, 10.0, 20.0, 30.0, 50.0 µg mL^{-1}, respectively.
